# Abstracts and Gleanings

**Published:** 1877-12-20

**Authors:** 


TYPHOID FEVER.

### ABSTRACTS AND GLEANINGS.

CASE OF EMPYEMA FOLLOWING PNEUMONIA—EARLY ASPIRATION—RECOVERY. The woman was admitted to the Bellevue Hospital in June, 1875, and had a certain amount of pus removed from the right pleural cavity. She left the hospital before recovery was complete, got married, and upon her return for examination exhibited a fine-looking boy about one year old. As far as her countenance indicated, she was in perfect health.

The history of this case during her stay in the hospital in 1875 was as follows:

She was 22 years of age, temperate, and gave no evidence of specific disease. Her mother died of cancer; otherwise her family history was good. One morning in June, 1875, she was seized with a chill which lasted for several hours, followed by fever and pain beneath the right breast of a severe, lancinating character, which continued throughout the night of the same day and up to the time of her admission to the hospital on the 22d of June—two days after she was attacked. On admission, her face was flushed, the pain in the side intense, the temperature 150° F. She was placed in bed, and a full dose of quinine administered. She had the physical signs of pneumonia at that time, such as dulness upon percussion, increased vocal fremitus, bronchial breathing, etc.

She passed through the pneumonia. On the 7th of July there was evidence of fluid in the right pleural cavity, and on the 20th of July a hypodermic needle was introduced and pus obtained. On the 23d of July, an aspirator needle was introduced, and sixteen drachms of pus removed. The expectoration was profuse and muco-purulent, but there were no evidences of pneumo-hydrothorax. On the 28th of July, the patient's friends, not wishing to have her "stuck with the needle," removed her from the hospital. At her examination, May, 1877, she stated that, after her removal from the hospital, she expectorated large quantities of purulent material, but did not have her chest aspirated. In May, 1877, there was found good resonance and good respiration over the entire right side of the chest. The woman had not noticed at any time that one side of the chest was smaller than the other. The case, then, was interesting as evidence of the value of early aspiration with the view of preventing retraction of the chest walls. What became of the pus which remained in the chest after the aspiration, and that which formed subsequently, was a question. It was thought probable that perforation of the lung took place, and that the pus was removed by expectoration.—*N. Y. Medical Record.*

---

TEETH AT BIRTH — EXTRACTION — HEMORRHAGE—DEATH.—The *Cincinnati Dental Register* supplies the following translation of an article written by Dr. Th. David, in the *Gazette des Hopiteaux:*

The child at birth was possessed of an ordinarily good constitution. Two days after it was noticed that the points of the two inferior central incisiors were visible about the margin of the gums. These teeth continued to emerge during the following days, and gradually loosened until they seemed to be supported only by a kind of neck, which allowed free movement in every direction. As they seemed ready to fall out, and troubled the child in nursing, the family physician, who was one of the masters in the Paris Hospital, believing their removal necessary, extracted them on the twentieth of January, that is to say, three weeks after the birth of the child. The operation was soon followed by severe hemorrhage, which could only be stopped by digital compression, which was continued during the whole night. The next morning the bleeding, which had for a short time subsided, recommenced, and continued the greater part of that day. Napkins were used to stanch the blood, which was in quantity really alarming. At two o'clock, one of our young and able practitioners was sent for, who immediately cauterized the anterior of the alveoli, from whence the blood came, with an olivary cautery about the size of a pea. This checked the hemorrhage for some hours, when it again commenced with the same intensity.

The next day (the twenty-second) cauterization was again resorted to in the same manner, and the flow of blood was stopped. Five days afterwards, however, it recommenced, necessitating a third application of the cautery. This last, unfortunately, resulted in the complete destruction of the gum and alveolar margin in the part corresponding to the two incisiors. During this operation. several hard and brilliant bodies were seen beneath the wound, which were none other than the crowns of several permanent teeth in process of formation.

Notwithstanding this last application of the cautery, the hemorrhage reappeared, and continued until the tenth of February, when we were again sent for, but the child was then dying.

Far from us the thought of criticizing the practice adopted in this case by our honorable *confreres.* We will, then, neither discuss the expediency of extracting these loosened teeth, nor the means employed in fighting against a hemorrhage of such an obstinate character. We are of opinion, that in this unfortunate affair, the extraction of the teeth only anticipated their spontaneous elimination, and we accept without reserve the explication arrived at by the council of physicians, viz: "That this child must be ranged in the category of those of Hemorrhagic Diathesis." We will add further that this disposition to hemorrhage seems to have originated in this child, and could not, therefore, have been foreseen, inasmuch as the parents showed no appearance of it, and the child was their first born.

Be this as it may, this case seems to us to contain certain lessons, and we have believed it our duty to cite it here, in treating the question of the precocious eruption of the temporary teeth, and the afflictions with which it may be complicated. But before speaking of precocious eruptions, we think we ought to determine the average time of normal eruptions.

First dentition, it is generally believed, commences about the sixth month after birth. All observers have been unanimous in giving this as the average time for the appearance of the first tooth; it is the opinion of Trouseau. But it will be seen after numerous researches on this subject, that we place the average time at the seventh month. Of five hundred births which we have observed, we have noticed the eruption of the inferior central incisors at the following periods:

| | | | | | |
|---|---|---|---|---|---|
| At birth | 1 | case. | At 7th month, | 105 | cases. |
| At 1st month | 2 | " | At 8th " | 88 | " |
| At 2nd " | 3 | " | At 9th " | 49 | " |
| At 3d " | 9 | " | At 10th " | 89 | " |
| At 4th " | 10 | " | At 11th " | 38 | " |
| At 5th " | 39 | " | At 12th " | 12 | " |
| At 6th " | 45 | " | At 2d year | 10 | " |
| Total | | | | 500 | |

—*Pacific Med. Jour.*

Successful Removal of a Large Fibroid Uterus with Both Ovaries.—Mr. Knowsley Thornton relates a case in which recovery took place after removal by gastrotomy of a large fibroid uterus with outgrowths, and both ovaries. The patient was 38 years old, married, but had never been pregnant. The tumor had been first noticed nearly three years before. The operation was performed on January 10th. In opening the peritoneum a coil of intestine was wounded by the point of the knife, but the wound was at once closed by a continuous suture of fine silk. The pelvic portion of the tumor could not be dislodged until the mass of the tumor was drawn out of the incision and used as a lever, by being pressed over the left iliac crest. This mass was then transfixed and ligated with two strong strings, and it was then cut off. Room was thus gained to get at the broad ligaments, which were transfixed and tied with double ligatures. The ovaries were then cut away. Finally the cervix was transfixed and tied, and the mass above it cut away. All the ligatures were cut short, and the abdomen was closed. The operation occupied rather more than an hour and a half. The ice-water cap was used on two occasions in the after-treatment, the temperature having risen to about 101 degrees. On the ninth day some red, offensive serum came away per vaginam, and this discharge continued till the eighteenth day. It then ceased, and at the same time pain was complained of in the right iliac region, and the pulse rose to 124. On examination by speculum a small slough was found plugging up the external os, and on pulling it away a quantity of fetid pus escaped. Convalescence then progressed favorably, and on the thirty-seventh day the patient was able to go out.

Mr. Thornton believes that this is the first successful case of removal of the uterus and ovaries, in which all the pedicles were tied with silk and left free in the peritoneum. He prefers this to the extra peritoneal method, thinking that it is attended by less danger of septicæma or of hemorrhage, experience having shown that the danger of hemorrhage when the clamp or wire separates is by no means small.—*Obstetrical Journal*, June, 1877.

Fecundation of the Hen's Egg in the Oviducts.—Dr. P. Tauber (*Naturh. Tidskr.*, R. 3, Bd. 10) divides this subject into two queries:

1. How long a time can a hen continue to lay fecundated eggs after separation from the cock, and how many fecundated eggs can such an isolated hen lay?

In the historical remarks with which the author introduces this section, he refers to the assertions of Fabricius ab Aquapendente, that one fecundation suffices for the remainder of the year, and Coste's doctrine that one fecundation is only effective for 5–7 eggs, which are laid in from 10–14, at the most 18 days after the hen's isolation—only dark yellow eggs of 15–35 millimetres diameter being capable of fecundation.

The author's investigations yielded the result that one treading suffices to fecundate 5–7, rarely 8 eggs, and that it was generally effective only till the 11th, rarely till the 18th day.

2. Is there in the hen's oviduct any particular place which can be rightly designated as a seminal receptacle?

The author here gives a general view of the hen's oviduct, in which, among other things, he denies the presence of glands. With regard to his especial subject, he divides it into the "tube" and the "infundibulum." He directs especial attention to the structure of the last-named portion, especially to a zone near the border of the infundibulum, which contains numerous excavations; these are, in his opinion, the special seminal receptacles. He finds that it generally takes the semen from 14 to 24 hours after the treading to arrive at this zone.

In contradistinction to Coste's idea that the fecundation occurs in the ovary, his investigations show that this probably occurs in the infundibulum, and that it is highly improbable that it can take place in the ovary. Especial attention is directed to the fact that on the rupture of the follicle in the infundibulum, the semen comes into direct contact with the germinal membrane.—*Nordiskt Med. Arkiv*, Bd. 9, Hæft I.

---

RHEUMATIC DISMENORRHEA.—Treatment during the menstrual week can have no effect beyond palliating the suffering of the patient temporarily. To become curative it must be extended through the interval, for the purpose of so changing the condition of the uterine structure and sensibility as to prevent the recurrence of the pain at the next period.

In the most common class of cases, in which the pain is severe and the flow scanty, I have for many years used successfully the following formula:

℞. Tinct. cimicifugæ, dr. iij
Tinct. stramonii, dr. ss
Vin. colchici rad., dr. ss Mix.

Take one drachm at each meal time in water.

If, by long continuance, or unusual susceptibility, the cimicifuga, causes dull headache, as is sometimes the case, either the dose should be lessened or the fluid extract of cypripedium may be substituted in its place. In the same manner, if the colchicum should cause disturbance of the bowels, its quantity must be lessened in proportion to the other constituents.

Another prescription with which I have succeeded in many instances, especially when the pain and soreness extended to the region of the ovaries, is as follows:

℞. Ammonia hydrochlor., dr. iij
Tinct. stramonii, dr. ss
Tinct. cimicifugæ rac., dr. iss
Syr. glycyrrhizæ, dr. ii Mix.

Take one teaspoonful at each meal time in a tablespoonful of water.

In a recent case characterized by extremely painful menstruation, accompanied by severe headache, continuing in less degree through the interval between the periods, some constipation and slight feverishness, I directed the following prescription:

℞. Acid. salicylici, oz. iij

Sodæ bicarb, oz. ij

Tinct. stramonii, oz. iv

Vin. colchici rad., oz. iv

Glycerinæ (pura), dr. i

Aquæ, dr. iij Mix.

Of this the patient took one teaspoonful before each meal time and at bedtime, in a little water. After she had taken the medicine, with the bathing and other hygienic regulations previously mentioned, a little more than three weeks, she menstruated freely, and, as she said, "without pain for the *first time* in several years."—*Dr. N. S. Davis, Chicago Medical Society.*

STRABISMUS.—There are two operations for strabismus: the simple tenotomy, and the tenotomy of one, and advancement of the opposite muscle. The simple tenotomy of the stronger, retracted muscle, is a comparatively slight operation, and especially since we perform it sub-conjunctivally, being borne, as a rule, almost without reaction.

The purpose of severing the muscle from its insertion upon the sclerotic, is to force it to attach itself further back. If immediately after the operation has been performed, we have not gained the desired effect, we may improve it by stitching the eyeball to the opposite corner. To judge of the effect we must keep in mind that immediately after the operation the patient should be able to move the margin of the cornea with the severed muscle respectively, to the caruncle, or to the outer commissure, according to the kind of strabismus which has been operated upon. If he can move the eye farther, we have not gained all that is necessary; if he cannot move it so far, we have done too much, and must reduce the effect by a suture, or the patient will, later on, squint towards the opposite side. This danger of converting, for instance, a convergent strabismus into a divergent one, is the reason why a conscientious operator never should operate on both eyes at one sitting. If we have not accomplished all we want by the operation on one eye, we may after some weeks correct the other one. Tenotomy is not generally so efficient in divergent, as in convergent squint. Where the simple tenotomy is not effective enough, we must resort to the advancement of the opposite muscle. The idea of this operation need not be explained.

The same that has been said concerning convergent and divergent strabismus is the rule for the much rarer forms of upward and downward strabismus. Cases, where the strabismus is caused by some other defect of the eye like leucoma cataract, apkakia, etc., have to be exempted from the foregoing remarks.

The operation for strabismus is so simple, that alone from an esthetic point of view, patients should undergo it, even if they would not gain more. Yet they can gain considerably more, and the more, the earlier the operation is performed. They may retain good, or comparatively good, vision, in an eye which without the operation, will get more and more useless, often so much so, that if the other eye later on is lost by accident, the patient is not much better than blind.—*Canada Lancet.*

---

SIGNS BY WHICH PHTHISIS IS RECOGNIZED IN ITS EARLIEST STAGES WITHOUT THE AID OF PHYSICAL EXAMINATION OF THE CHEST.—(*The Medical Record*, Sept. 1, 1877.)

1. Retraction of the skin over the cheeks.

2. Cerulean hue of the sclerotic, due to anæmia of the conjunctiva.

3. Atrophy of the lips, of the ears, and a thin, pinched appearance of the nose. Whenever the skin closely covers cartilages, as in the ears and nose, a showing through, as it were, of the cartilaginous framework is one of the earliest signs of loss of flesh.

4. Palor of the cheeks and face as compared with each other and with the malar surfaces.

5. Dilatation of the nostril upon the affected side. This is the case in all pulmonary affections, but especially in the earliest stages of phthisis.

6. The respiration is invariably accelerated, and the disturbance affects expiration as well as inspiration. In certain nervous disturbances the respiration is accelerated, but it is the inspiration only which is at fault.

7. Sinking of the clavicle more upon the affected side than upon the opposite, and giving the appearance of having a very long neck.

8. Great hyperæmia of the pillar of the fauces, present long before the pulmonary disease manifests itself, and continuing until pus is expectorated. When purulent expectoration is established, decomposed pus irritates the throat, and then the other parts usual become hyperæmic.

9. Intense congestion of the throat, early hoarseness and vomiting are unfavorable symptoms, and indicate enlargement of the bronchial glands. This vomiting is caused by pressure upon the pneumogastric by the enlarged glands. A large proportion of phthisis cases will tell of having had sore throat for a number of years previous to the development of any chest symptoms.—*Clinic.*

INFLUENCE OF DRINKING-WATER ON THE BILIARY SECRETION.—Zawilski placed glass canulæ in the ductus choledochus of rabbits, closed the intestine, sewed the canula in the abdominal wound, and either collected the bile secreted, drop by drop, in vessels, in order to estimate the entire amount of bilious fluid in a certain interval, together with its dry residue and the amount of its constituents, organic and inorganic, or he allowed the bile to collect in a vertical glass tube, in order to measure the force of its secretion. Researches were instituted upon animals simply tied, in comparison with those in whom a wound had been made in the œsphagus, through which, by means of a catheter, ordinary water, carbolic acid, oxygen, or ozonized water in small quantities were introduced. The results were as follows:

1. In animals who have fasted for fifteen hours, a decided diminution in the secretion of bile is noticeable. At the same time, the percentage of fixed substances diminishes, the bile being more watery.

2. Water, when absorbed from the stomach, increases immediately the secretion of bile, while, at the same time, the quantities of solids is augmented; that is, a larger quantity of concentrated bile is poured out. The amount of gas contained in the water is a matter of indifference, provided the gases do not distend the stomach, and thus act mechanically as a hindrance to the exit of the bile. The secretion of bile is so much the more abundant the longer the water is supplied. Frequent and small amounts of water exercise the most favorable influence upon the biliary secretion. By the administration of water, not only is the quantity of bile increased, but the pressure under which it is secreted is rendered greater, so that obstacles which, under ordinary circumstances, would hinder the flow of this fluid are overcome.—*The Doctor*, October 1, 1877.

A FEMALE CHEMIST.—A Russian journal tells the following incident:

A young Russian has for some time been prosecuting his chemical studies at the University of Leipsic, with unusual zeal. The young man, of aristocratic exterior, made friends of all who came in contact with him. Recently, he passed a most brilliant examination, which was rewarded with the dignity of a master of arts. Soon after, a young lady called on one of the most prominent professors of the university, addressing the *savant* in the following words:

"I desire, professor, before I depart from Leipsic, to express to you my hearty thanks."

The professor, perfectly astonished, observed, "thanks, but for what?"

"Listen, sir. I was married to old Prince ——. My husband died some years ago. He died insolvent, so that I was left even without the daily bread. I resolved to seek the necessary means of subsistence in science."

The professor then interrupted her, saying: "Yes, most gracious lady; nevertheless, I cannot see why you should address any thanks to me."

The lady continued: "Observe, then: it is now more than three years that here in Leipsic I have been a student. The student who lately passed the examination, and whom you considered worthy of distinction, is none other than myself."—*The Sanitarian.*

LEMON-JUICE IN RHEUMATISM.—Dr. A. H. Chandler, in *Canada Lancet*, says: In advocating the lime or lemon-juice treatment, the author cannot, of course, presume to suggest anything novel; but, he does venture on claiming originality, with regard to the *largeness* and *frequency* of the dose, and hesitates not to offer it, when so given, as a veritable specific in this, not seldom, treacherous and intractable malady. Without regard to the condition of the bowels, unless previously much constipated, I usually begin with at least ten ounces of lime-juice, increasing rapidly up to eighteen or twenty-four in the twenty-four hours—from half an ounce to an ounce, or more every hour, with not less than double or treble the quantity of cold, soft water—usually diluted and sweetened, however, to the patient's taste. Very often, on the second day, the amendment is decided, and the disease, in acute cases, more particularly sthenic or asthenic, generally subsides on the fourth or fifth day of treatment. One grain of opium is usually given, with or without lead, and tannin, night and morning, in order to restrain the bowels, which the juice has a tendency to relax. The first effect of such heavy doses is the rapid diminution of joint swelling, and diminished perspiration, together with steady falling of pulse, the latter often quite slow with a slight tendency to syncope, the majority of the cases requiring quinine, and supporting food about the sixth or seventh day, when convalescence advances rapidly.

---

JAPANESE THERAPEUTICS.—Dr. G. Maget has furnished on this subject some interesting notes. General and local abstraction of blood is rejected by Japanese practitioners, on the plea that the blood is too precious a fluid to be thus wasted. Febrile affections are chiefly treated by copious draughts of warm water, under the idea of relaxing the pores which have been constricted by cold. Calomel is a very favorite remedy, and is better supported by the natives than by Europeans. Blisters are popular; they are made with the powder of the *Pagara piperata*, spread on a rice plaster. Moxas are very frequently had recourse to as derivatives. Their active principle is extracted from the Artemesia Japonica. In less urgent cases acupuncture is employed, as in China, to replace the Moxa, chiefly in abdominal affections, twenty needles being inserted in each flank. Shampooing is a very popular mode of treatment in rheumatic affections and certain cases of nervous debility; also, as an hygienic precaution against the fatigue of a long journey or of protracted labor. The Japanese ladies are so extremely modest that they employ none but the blind to shampoo them. Syphilitic affections are common, and are combatted by cinnabar (red sulphuret). The soft sore is much more frequent than the hard one. In Japan, as elsewhere, the introduction of the evil is attributed to a neighboring country.—*Medical Examiner*, Nov. 1, '77.

---

GELSEMINUM AND QUININE.—Dr. R. B Trezevant, Desarc, Ark., suggests, in *The Medical Brief*, the following formula:

℞. Ferri sulph.. } of each 1 drachm.

Quinæ sulph, }

Acid nitric . }

Aquæ. .............. 8 ounces.

Mix the iron and acid, and as soon as fumes cease to rise, add one ounce of the water; in this dissolve the quinine, then add the other seven ounces of water.

S. Give one teaspoonful in wineglassful of water three times a day.

In conjunction with this, I usually prescribe once a week, a mercurial or vegetable purge.

In regard to the use of gelseminum, it is almost a rule with me in the many forms of continued fever, especially when there is nervous derangement, to give quinine in the following manner:

℞. Quinæ sulph, 16 grains.

Spt. eth. nit., 4 drachms.

Tr. gelsemini, 2 drachms.

Aquæ, 4 ounces.

S. Give one tablespoonful every three hours.

This makes a perfect solution, and as a rule has, in my hands, rapidly reduced febrile symptoms, curbed all restlessness — so much so as to lead some patients to think they had taken an opiate.

WHITLOW OF THE THUMB.—The first phalanx of the thumb of this patient presents two incisions. The first, located upon the inner side, was made some days ago without procuring any relief. The swelling and pain having increased, the man entered the wards of M. Verneuil. His interne made a deep incision in the median line. The pain soon disappeared and the phlegmon has entered upon the road to resolution. Apropos of this case, M. Verneuil remarked that in whitlow in general, the incision ought never to be made except in the median line. Lateral incisions not only expose to wounding the arteries and nerves, with their consequences, that is to say hemorrhage and temporary anæsthesia of the organ, but besides it is seldom that they afford relief to the patient. Once again, incisions into the median line ought always to be preferred, for this double reason, that they expose to no accident, and are much more efficacious.—(Canadian Journal of Medical Science, from *Revue de Therapeutique Medico-Chirurgical.*

ARSENICAL ANTIDOTES(*Rep. de Pharm.*, 1876. *Boston Medical and Surgical Journal*).—From some late experiments, Rouyer has found that, although the freshly precipitated sesqui-hydrate of iron is an antidote for arsenious acid, it has no effect in counteracting the action of sodic arseniate or potassic arsenite (Fowler's solution), but that a mixture of the solution of sesqui-chloride of iron and the oxide of magnesium will counteract the effect of these salts as well as the arsenious acid itself, and hence this mixture is always preferable to the hydrate in cases of arsenic poisoning. The officinal solution of the sesqui-chloride of iron should be first administered, and fifteen minutes afterwards the magnesia oxide, given in the proportion of four grammes of the latter to one hundred cub. cent. of the former. In one hour after the administration of the antidote, a cathartic should be given. The ingestion of acid drinks and lemonade should be avoided during the entire treatment, since the compound formed by the union are soluble in acids.—*Clinic*

THE EFFECTS OF IRON UPON THE BLOOD.— Dr. Nasse, of Marburg, relates his experience in the *Cent. Blatt. f. Med. Wissenschaften.* For 77 days he fed a dog, weighing about eight kilogrammes, upon bread and potatoes, to which he had added iron; for 25 days he gave it one gramme of lactate of iron daily, and for the remaining 62 days he gave 1.2 grammes of oxide of iron daily. The weight of the animal rapidly increased, and at the end of the experiment it had gained one kilogramme. The specific gravity of the blood increased from 1052 to 1060.8, that of the serum, however, remained unchanged. The proportion of iron increased from 0.477 to 0.755 per thousand parts. In seven other dogs of the eight experimented upon, the amount of solid constituents and the specific gravity both increased. The increase of the former can be due only to an increase of the blood corpuscles.—*Allgemeine Medicinische Central-Zeitung*, No. 76, 1877.

SYPHILITIC FATHERS AND HEALTHY CHILDREN.—Dr. Guntz, of Dresden, reports six cases in which the fathers of healthy children were the subjects of latent syphilis. In each of these cases at least two years had elapsed between the latest manifestations of syphilis and marriage. In several cases, the first child was syphilitic, the second healthy. In all of the cases, within a few years of the birth of their children, the deeper syphilitic lesions manifested themselves in the persons of the fathers, all of whom had had good reasons, among others the birth of healthy children, to suppose that the disease was eliminated from their system.—*Vjhrschr. f. Dermatol. u. Syph.*, and *Phila. Med. Times*, July 7, 1877.

CHENOPODIUM ANTHELMINTICUM FOR ANASARCA.—Dr. Samuel R. Burroughs, of Houston, Texas, in a report on the "Indigenous Medical Resources of Texas," published in the *Transactions of the*

*Texas State Medical Association*, 1877, makes the following foot-note on page 108: "The chenopodium anthelminticum root, split or cut to pieces, and put into an ordinary bottle filled with vinegar, to which has been added two or three drachms of carbonate of iron, will relieve anasarca when a sequel to scarlatina, or when dependent on any functional derangement."

Incidentally, Dr. B. remarks that a decoction of this plant, (which is abundant in Texas, and commonly known as Jerusalem oak, wormseed, etc.) together with the cooked plant, mixed with meal, is said to be also a good remedy for chicken cholera.—*Va. Med. Monthly*.

---

TREATMENT OF BOILS, CARBUNCLES, ETC.—Dr. E. Bodman, of Sidney, Ill., sends us the following note:

Years ago I was in the habit of treating boils, carbuncles, etc., with a strong solution of permanganate of potassa in the following manner: I laid the boil open to its bottom with a free incision, and filled it with linen lint, fully saturated with the solution. The results were quite satisfactory, but for some time past I have employed the following treatment, which has been completely successful: I take equal parts of carbolic acid, glycerine and water, and inject a few drops (5 to 30) with the hypodermic syringe into the tumor in its deepest part. One injection is usually enough, if applied in the early part of the disease. The tumor at once shrinks away with slight pain or soreness, leaving but little disposition to successive crops. If this mode of treatment has been in use by others, I have never heard of it.—*Boston Journal of Chemistry*.

---

MORPHIA IN CATHETERISM.—Dr. W. Faulkner (in *Brief*) says: "Some may regard it as a very easy and simple thing to pass the male catheter, and think a failure to do so necessarily to depend on the condition of the parts; while, *I think*, the facts justify me in saying that it is an operation requiring experience, tact and skill, and the strong probabilities are that we often fail for want of experience in the use of the instrument. I have been well pleased with the hypodermic use of morphia as a preparatory measure to the use of the catheter; and I have for some years regarded it not only as highly proper to aspirate or puncture the bladder when I could not pass the catheter, but have felt it to be a duty and an obligation I owe to my patient thus to protect him from the consequences of over-distension and blood-poisoning.

---

BREWERY GRAINS AS FOOD FOR COWS.—The custom of feeding brewery grains to cows to increase the flow of milk is very common in all large cities. The result is an excess of quantity for the time being, with a very decided deterioration in quality; but, sooner or later this food, when used in considerable quantities, produces a poisonous effect on the animals, and renders the milk wholly unfit for use. The cows, if fed on grains alone, become covered with sores and eventually die. The poisons that are used in the manufacture of malt liquors, such as sulphuric acid, cocculus indicus, opium, copperas, alum and strychnine, naturally settle (especially the dregs of them), in the grains. This furnishes a clue to the increased infant mortality in large cities.—*Canada Lancet.*

---

ARSENIC IN ALBUMINURIA.—In *The Practitioner* (June, 1877,) a case of albuminuria of nine years' standing is reported as cured by arsenic in the form of Fowler's solution, three drops at meal time. It is supposed that the remedy is adapted to that form of the disease which proceeds from pancreatic derangement. As a healthy pancreatic secretion serves to convert coagulated albumen into a soluble form before finally digesting it. If not so converted, the albumen is absorbed from the intestines and passes off by the kidneys, constituting albuminuria.

---

HYDROPHOBIA DURING PREGNANCY.—The following case has been communicated to the Academy of Medicine of

Paris, by Professor Borley, for M. Congier, of Bagnieres: A woman, aged 42 years, when about eight months pregnant was bitten on the hand by a cat. She was confined in due course. About six weeks after, and ninety days after the bite, the lochial discharge became altered. Shortly, marked simptoms of hydrophobia set in, and the woman died in five days. The child was healthy.—*The Doctor*, October 1, 1877.

---

Fracture of the Femur—Buck's Method.—Dr. F. H. Hamilton, in Bellevue Hospital, thus describes Buck's treatment of fractured femur: Dr. Buck has done a great deal for the treatment of these fractures, but the various improvements which have been adopted in its most approved form have been suggested by so many surgeons that I think it is hardly just that it should be called by his name, and I would suggest the "American plan" as a more appropriate title. You observe its prominent points: The long splint, with its lower extremity fitted into a light wooden frame-work to hold it steady, and its upper portion bound to the side of the chest by a wide roller-bandage; the foot-piece (to which the weight is attached by the cord passing over a pulley) sufficiently wide to prevent any pressure being made upon the external or internal malleolus; the adhesive strips extending up to the knee, and covered by a roller to keep them in position; the four short side-splints about the thigh, covering the seat of fracture; and, lastly, the foot of the bed elevated four inches above the floor, for the purpose of making counter-extension. The adhesive plaster should not pass above the knee; for if it reaches higher than that, it will be likely to do more harm than good, by involving some of the muscles which are attached to the upper fragment of the femur. For the four independent side-splints, within the long one, we are now in the habit of using felt, because it is a light material, and when once molded to a part retains its shape permanently. They are kept in position by a bandage, and can be removed at pleasure for the purpose of examining the seat of fracture, or for any other reason that may necessitate it. They are extremely useful in preventing looseness of the limb. As a general rule, I regard the long splint as the most essential requisite for making a straight thigh, and it acts in two ways: *first*, by preventing eversion; and, *second*, by keeping the whole body straight. In its simplicity and efficiency it is far superior to the plaster-of-Paris bandage. Theoretically, the latter, after being once applied, is supposed to remain *in situ* until the case is discharged cured; but practically, it is found to get loose in a week, and in two weeks it becomes positively necessary to remove it, and apply an entirely new dressing, which involves no inconsiderable amount of labor. This, of course, has to be repeated about every fortnight, until the end of the treatment. Here is a little boy, upon whom the plaster was applied only a few hours ago; and, though it was very carefully and thoroughly done, you will observe that I can already get my hand underneath the part of the bandage which passes around his body. In the course of a week the whole will be so loose as to be of no practical use whatever.

In all the cases which I have shown you, there will probably be some shortening, varying from three-eighths to one half of an inch; for in fractures of the femur, more or less shortening is the rule, and not the exception. Some writers would have us believe that naturally in about every third man one lower extremity is longer than the other, but this is certainly not the case; for were it so, this disparity would very frequently be corrected by the occurrenee of a fracture. In reality, however, I find that in about nine out of every ten cases one limb is slightly shorter than the other after my treatment for fracture.—*Med. Times*.

---

For Salivation.—*Bromo-Chloralum*' (Tilden & Co.)

IODOFORM IN TYPHOID FEVER.—Letter from B. D. Keator, M. D., Tolono, Ill., Oct. 27th, 1877:

"I called the attention of the profession, a short time since, to the value of *Iodoform* in Dysentery. Its benefit in that diseaee, especially the chronic form, is being fully confirmed. I now wish to call attention to its value in *Typhoid fever.* It acts, apparently, by healing the diseased intestinal glands. Give the patient the usual care, in alimentation, sponging, etc., and as a medicine, throughout the continuance of the disease, one-half grain Iodoform, every four hours, in pills or emulsion. So used, I believe it to be in advance of any treatment hitherto employed. No trouble with diarrhœa will usually be had; if so, a little opium combined with it will soon remedy it. When necessary to move the bowels, use hourly a teaspoonful of oil, or an enema of tepid water."—*Jour. Mat. Med.*

---

QUINIA IN SMALL DOSES IN MALARIAL DISEASES.—Dr. Charles R. Greenleaf, U. S. A., gives the formula as one coming from an army surgeon, whose name and present locality we now forget. If it accomplish what is claimed—the prevention of return of intermittent attacks by so small a dose as two grains of quinia—then it will save an immense expense, and obviate the unpleasant effects which the larger doses often produce. We have not met the formula before, though it may not be new to others:

R. Quinæ sulph., grs. ij
Acid. citric., grs. iij
Rub fine together and dissolve in aqua, dr. ss
Potass. iodid., grs. iij
Aquæ, dr. ss

Then mix the two solutions together, and administer the whole quantity at a dose.—*Amr. Prac.*

---

SOLUTION OF QUININE FOR HYPODERMIC USE.—Dr. James E. Morris, of Belleville, Texas, sends the following formula for a solution which in his practice has operated satisfactorily: Bromide of quinia dissolved in alcohol, grain for minim; to this solution water can be added to any dilution desired. It acts promptly and leaves no scar. One of the advantages claimed for this solution is that the alcohol prevents the development of fungi. It is readily absorbed, usually, and has a peculiar quieting effect upon the nervous system.—*Clinic.*

---

PREVENTION OF LEAD POISONING.—Some lead-workers have lately tried a careful washing of the hands in petroleum as a preventive of lead-poisoning. Three washings a day are said to be suficient to prevent all serious danger of lead-poisining. The success obtained by these men has been such that they have recommended a trial of petroleum as a guard against poisoning by salts of copper or mercury, where these are employed in trade.—*Brit. Med. Journ.*, Sept. 29, 1877.

---

KEROSENE LINIMENT.—Dr. Hobbs, in *Louisville Med. News*, recommends the following as a very superior liniment:

R. Kerosene oil.................dr. ij;
Tinct. Opii...................oz. iv;
Tinct. Arnica.................oz. v;
Tinct. Stramonii..............oz. iv;
Ar. Spts. ammon...............oz. vi;
Spts. Camph...................oz. v;
Ol. origan....................oz. iv;
Chloroform....................oz. iij. M.

S. Rub in twice during the twenty-four hours, or *pro re nata.*

---

Dr. H. A. Martin (Bost. Med. Jour., Feb. 1, '77), says that, during the sixteen years in which he supplied humanized vaccine virus, he was continually troubled by the complication of erysipelas. Since he has supplied only the bovine virus he has had no complaint of erysipelas.—*Dht. Med. Jour.*

---

SCLEROTIC ACID.—This is the latest therapeutic novelty. It is said to be the active principle of ergot, and has been isolated by Dragendorf. As yet its price is high, $20 per ounce; but the dose is small, gr. 1-12-1-16, by hypodermic injection.—*Med. and Surg. Rep.*

