# Important Errors

**Published:** 1878-01-20

**Authors:** 


### IMPORTANT ERRORS.

On page 325, December number of our journal, in an article copied from another journal, on Rheumatic Dysmenorrhea, two of the formulæ are wrong, and it would be well for each subscriber to turn to it and correct with his pencil, to read as follows:

R.—Ammonia hydrochlor.................dr. iii.

Tinct. Stramonii......................dr. ss.

Tinct. Cimicifugæ.

Tinct. Glycyrhizæ................aa. oz. iss.

R.—Acid Salicylic.......................dr. iii.
Tinct. Stramonii.......................dr. iii.
Soda Bicarb...............................dr. ii.
Vi um Colchici..........................dr. iii.
Glycerinæ...................................oz. i.
Aquæ ........................................oz. iii.

